# Around the Hospitals

**Published:** 1894-04-07

**Authors:** 


### Around the Hospitals.

Notice to the Managers of Institutions.—Special space is now reserved for the insertion of notices of hospital meetings and festivals. The Editor requests that all notices may reach The Hospital Office, 428, Strand, at least one week before the date of each meeting.

**Hastings and East Sussex Hospital.**—A most satisfactory report of this hospital was given at the annual meeting, a far higher number of in-patients and out-patients than usual having been treated during the past twelve months. The children's ward, furnished by Mrs. Tubbs with sixteen cots and other requisites, has proved a valuable addition to the comfort of many little sufferers. The donations received during the past year have been large, one including £500 given anonymously and £100 from Colonel Hankey. Many of the Governors have increased, and in some instances doubled, their subscriptions. A very successful bazaar, held under the superintendence of the Matron, Miss Louth, realized £150. The total number of patients treated during last year was 3,685—558 in-patients and 3,127 out-patients. Forty-seven in-patients remained on the books at the close of the previous year, making the number of patients actually admitted 511. As regards the financial record of the hospital, it may be considered most satisfactory. The asse s over liabilities on January 1st, 1893, as per last balance-sheet, amounted to £14,190 16s. 4d., to which has to be added legacies received during the year, £54, making altogether £14,244 16s. 4d. From this has to be deducted the excess of expenditure over income (exclusive of legacies for the year 1893) amounting to £249 8s., thereby reducing the assets at December 31st, 1893, to £13,995 8s. 4d.

**The General Hospital, Birmingham.**—Subscribers to this most useful institution still continue to show no increase of numbers, which is to be regretted, seeing that the number of patients treated grows steadily, and the expenses have been added to owing to the wise provision of tea, sugar, crockery, cutlery, and soap by the hospital instead of by the patients as formerly. The legacies during the past year have been exceptionally small, and the overdraft against the hospital amounts to £305. The Samaritan Fund finds plenty to do. Clothing has been provided for more than 100 patients, cab and railway fares paid, and 274 patients have been sent to convalescent homes through its agency. The in-patients at the infirmary during 1893 amounted to 15,873, the out-patients to 58,181. The ordinary expenditure was £17,085, the income £13,112. The average cost of each in-patient was £2 10s. 10d.; the cost per occupied bed per annum was £71 10s. 6d.

